# The associations of psychopathology and metabolic parameters with serum bilirubin levels in patients with acute-episode and drug-free schizophrenia: a 5-year retrospective study using an electronic medical record system

**DOI:** 10.1186/s12888-024-05862-5

**Published:** 2024-05-29

**Authors:** Yinghan Tian, Cheng Yang, Lewei Liu, Xin Zhao, Haojie Fan, Lei Xia, Huanzhong Liu

**Affiliations:** 1 Department of Psychiatry, Chaohu Hospital of Anhui Medical University, 64 Chaohu North Road, Hefei, 238000 Anhui Province P. R. China; 2 Anhui Psychiatric Center, Anhui Medical University, Hefei, Anhui Province P. R. China; 3 Anhui Provincial Key Laboratory for Brain Bank Construction and Resource Utilization, Anhui Medical University, Hefei, Anhui Province P. R. China; 4 Department of Psychiatry, School of Mental Health and Psychological Sciences, Anhui Medical University, Hefei, Anhui Province P. R. China

**Keywords:** Bilirubin, Schizophrenia, Psychopathology, Glycolipid metabolism, Oxidative stress, Biomarker

## Abstract

**Background:**

The oxidative system plays an important role in the pathogenesis of schizophrenia. Inconsistent associations were found between hyperbilirubinemia and psychopathology as well as glycolipid metabolism in patients with schizophrenia at different episodes. This current study aimed to examine these associations in patients with acute-episode and drug-free (AEDF) schizophrenia.

**Methods:**

This is a retrospective study using 5 years of data from May 2017 to May 2022 extracted from the electronic medical record system of Chaohu Hospital of Anhui Medical University. Healthy controls (HCs) from the local medical screening center during the same period were also included. Participants’ data of the bilirubin levels [total bilirubin (TB), conjugated bilirubin (CB), unconjugated bilirubin (UCB)], glycolipid metabolic parameters and the score of the Brief Psychiatric Rating Scale (BPRS) were collected.

**Results:**

A total of 1468 case records were identified through the initial search. After screening, 89 AEDF patients and 100 HCs were included. Compared with HCs, patients had a higher CB level, and lower levels of glycolipid metabolic parameters excluding high density lipoprotein-cholesterol (HDL-C) (all *P* < 0.001). Binary logistic regression analyses revealed that high bilirubin levels in the patients were independently associated with higher total and resistance subscale scores of BPRS, a higher HDL-C level, and lower total cholesterol and triglyceride levels (all *P <* 0.05).

**Conclusion:**

Bilirubin levels are elevated in patients with AEDF schizophrenia. Patients with high bilirubin levels have more severe psychopathology and relatively optimized glycolipid metabolism. In clinical practice, regular monitoring of bilirubin levels in this patient population should be carried out.

**Supplementary Information:**

The online version contains supplementary material available at 10.1186/s12888-024-05862-5.

## Introduction

Approximately 1% of the worldwide population is affected by schizophrenia [1], while the China Mental Health Survey showed that the lifetime prevalence of schizophrenia in China is 0.6% [2]. Schizophrenia is often combined with hyperbilirubinemia, which is usually considered a sign of liver or hemolytic diseases [3, 4]. The prevalence of hyperbilirubinemia is about 25% in patients with schizophrenia, which is 2–3 times higher than in the general population [5].

Bilirubin is the end product of heme metabolism in human red blood cells, and comes in two forms: conjugated bilirubin (CB) and unconjugated bilirubin (UCB) [6]. On the one hand, a study reported that bilirubin had a strong antioxidant effect at a concentration below 100 nanomoles per liter (nM), which can protect nerve cells from oxidative stress damage [7]. On the other hand, high bilirubin levels can exhibit significant pro-oxidant and neurotoxic effects, ultimately leading to neuronal necrosis or apoptosis [8, 9]. The dysfunction of the oxidative system may be involved in the pathogenesis of schizophrenia [10]. In both animals and humans, higher bilirubin levels have been shown to be strongly associated with a greater risk of schizophrenia and more severe psychiatric symptoms. For example, an animal study showed that Gunn rats, a model of hyperbilirubinemia, have remarkably schizophrenic-like behaviors [11], and a cohort study with 21-year follow-up found that newborns with hyperbilirubinemia had an increased risk of developing schizophrenia in adulthood [12]. Moreover, Miyaoka et al. reported that levels of biopyrrins, oxidative products of bilirubin, are positively correlated with the score of Brief Psychiatric Rating Scale (BPRS) in patients with chronic schizophrenia [13]. Additionally, previous studies found that there were significant differences in UCB levels among three groups (schizophrenia > schizoaffective disorder > bipolar disorder), which indicates that UCB may be a potential biomarker in schizophrenia [14–16]. Therefore, given the complex mechanisms of action of bilirubin, it is important to reveal the characteristics of bilirubin and its relationship with psychopathology in patients with schizophrenia.

In the general population, bilirubin levels were negatively correlated with total cholesterol (TC), triglyceride (TG), and fasting blood glucose (FBG) levels, which have been repeatedly confirmed in previous meta-analysis, cohort study, and national survey [17–19]. Similarly, in patients with schizophrenia, CB level was negatively correlated with the levels of TG and FBG, which has been shown to be a valid predictor of risk for metabolic syndrome (MetS) [20].

The differences in bilirubin levels in patients with schizophrenia across studies may be due to the heterogeneity of the subjects studied (e.g. gender, intake of antipsychotics, and different periods of illness). For example, in a previous cross-sectional study, bilirubin levels were higher in males with schizophrenia than in females [21]. Christian et al. found that patients with schizophrenia had significantly lower bilirubin levels after 2 and 4 weeks of antipsychotic treatments [22]. Moreover, both case-control and prospective studies showed that patients with acute-phase schizophrenia had higher bilirubin levels than those with remission-phase schizophrenia [23, 24]. Therefore, this current study aimed to examine (1) the levels of bilirubin in patients with acute-episode and drug-free (AEDF) schizophrenia and their associations with psychopathology, and (2) the associations between bilirubin levels and glycolipid metabolism parameters in patients with AEDF schizophrenia.

## Methods

### Study design and participants

This is a retrospective study using 5 years of data from May 2017 to May 2022 extracted from the electronic medical record system of Chaohu Hospital of Anhui Medical University. Patients were included, if they met the following criteria: (1) Han Chinese, aged between 18 and 65 years; (2) diagnosed as schizophrenia, based on the Diagnostic and Statistical Manual of Mental Disorders, 5th edition (DSM-5) using a structured Clinical Interview, and were in an acute-episode as defined by BPRS score ≥ 40 [25]; (3) without antipsychotic treatments for the last 1 month [26]. Patients were excluded who had (1) any other psychiatric disorders; (2) organic brain diseases, hypertension, diabetes, hyperlipidemia, immune system diseases, hepatic diseases or other serious physical diseases; (3) or were pregnant or breastfeeding women; (4) recently used any anti-inflammatory drugs. The HCs were recruited from the local medical examination in the same period, who were Han Chinese, aged between 18 and 65 years, and had no personal or family history of psychiatric diseases.

In accordance with the Declaration of Helsinki, all participants and their guardians signed an electronic informed consent via smartphone. The protocol of this retrospective study was approved by the Medical Ethics Committee of Chaohu Hospital of Anhui Medical University on 13 October 2022 (202210-kyxm-015).

### Data collection measurements

All participants’ socio-demographic and clinical data were collected through the electronic medical record system, including age (years), sex (male and female), age of onset (years), duration of illness (months). Body mass index (BMI) was calculated as weight (kg)/height (m)^2^. Blood samples from patients were collected between 06:00 and 07:00 AM after an overnight fast. And bilirubin [total bilirubin (TB), CB, UCB], and glycolipid metabolism parameters [TC, TG, high density lipoprotein-cholesterol (HDL-C), low density lipoprotein-cholesterol (LDL-C), FBG] were measured. Psychiatric symptoms of the patients included in this study were assessed using the Chinese version of the 18-item BPRS [27, 28]. Each item was evaluated on a seven-point scale, ranging from “0 = not present” to “7 = extremely severe”, with a higher total score indicating more severe symptoms [29]. The scale consists of five subscales: affect subscale (items 1, 2, 5, and 9), negative symptoms subscale (items 3, 13, 16, and 18), positive symptoms subscale (items 4, 8, 12, and 15), resistance subscale (items 10, 11, and 14), and activation subscale (items 6, 7, and 17) [30].

### Statistical analysis

The continuous and categorical variables were described as Mean ± standard deviation (SD) and frequency distributions (%), respectively. The Kolmogorov–Smirnov one-sample test was used to test the normal distribution of continuous variables. Patients were divided into low and high serum bilirubin groups using the 50th percentile of total TB, CB, and UCB, respectively [20]. Socio-demographic and clinical characteristics were compared between groups using independent samples t-test, Mann-Whitney *U* test, and Chi-square test as appropriate. Binary logistic regression models (Forward: LR) were used to examine the independent correlates associated with bilirubin levels, with bilirubin levels (categorical) as the dependent variables, and the variables which were significant in univariate analyses (*P* < 0.05) as the independent variables, as well as age and sex as the control variables. The correlations between bilirubin levels (after ln-transformed) and other clinical data, as well as metabolic parameters in the patients group were examined with Pearson or Spearman correlation analyses. Then multivariate linear regression models (Forward: LR) were used to examined any significant correlations (*P* < 0.05) in correlation analyses. Statistical Product and Service Solutions (SPSS) version 23.0 (SPSS incorporated, Chicago, Illinois, United States of America) was used for statistical analyses. The *P* - values were set as two-tailed *α* = 0.05.

## Results

### Case record selection and participant characteristics

**Fig. 1:**
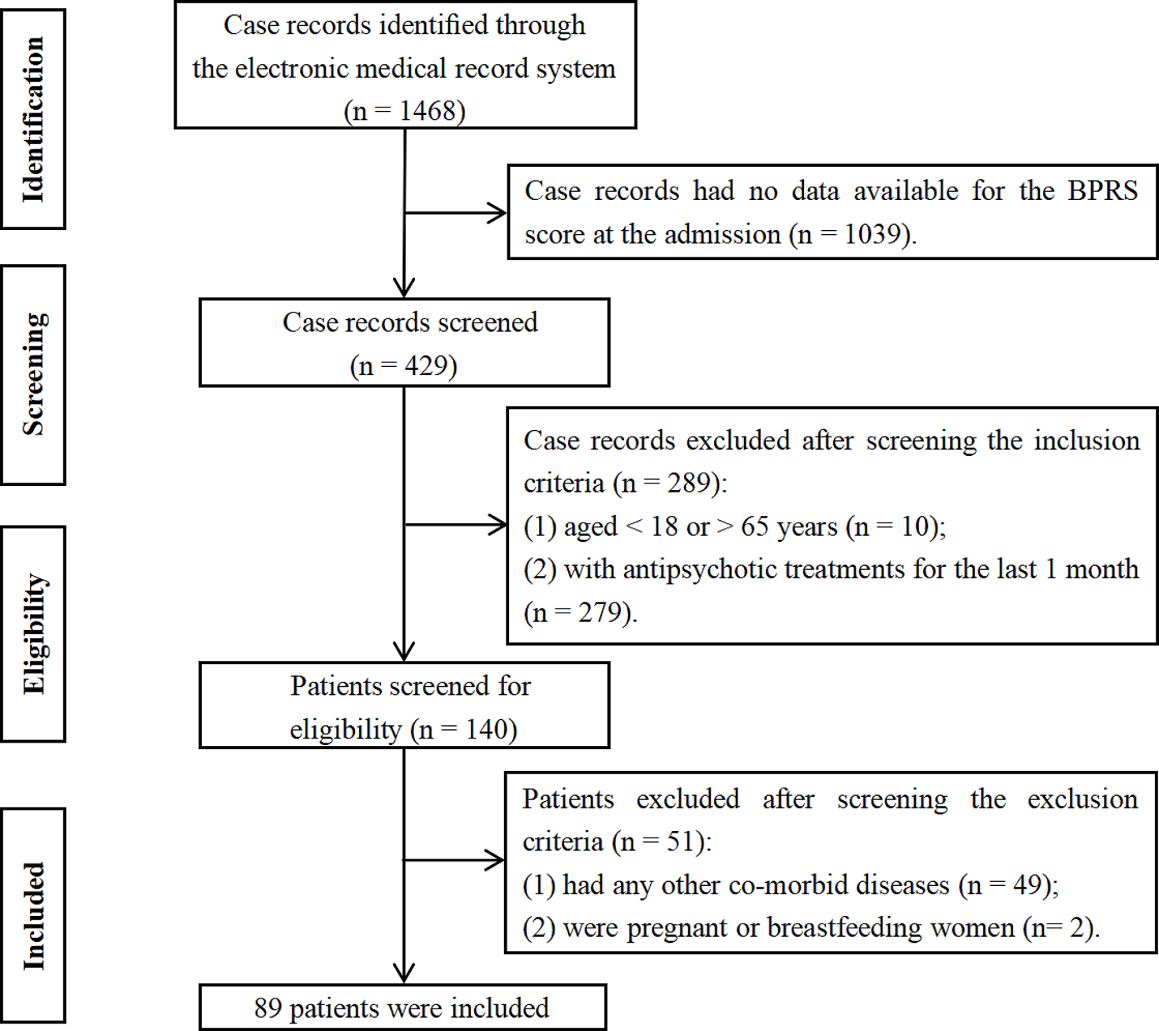
Flowchart of the case records selection

A total of 1,468 potentially relevant case records were initially identified (Fig. 1). After screening according to the inclusion (1,328 excluded) and exclusion (51 excluded) criteria, 89 patients with AEFD schizophrenia were included. And 100 HCs from the local medical examination were also included. In this study, the mean age of patients was 38.92 years (SD = 13.75), and 42.7% were male. The mean age of HCs was 35.35 years (SD = 6.95), and 42.0% were male. There were significant differences in BMI between groups (all *P* < 0.001) (Table 1).

**Table 1:** Socio-demographic and clinical characteristics of patients and healthy controls

Variables	Patients (*n* = 89)	Healthy controls (*n* = 100)	t/Z/χ2	*P*
Age (years)	38.92 ± 13.75	35.35 ± 6.95	-1.574^a^	0.116
Male (%)	38 (42.7)	42(42.0)	0.009	0.923
BMI (kg/m^2^)	21.88 ± 1.73	23.27 ± 3.06	3.915	**< 0.001**
Age of onset (years)	34.06 ± 14.09	-		
Duration of illness (months)	57.32 ± 86.54	-		
TB (µmol/L)	17.13 ± 9.22	14.28 ± 4.19	-1.223^a^	0.221
CB (µmol/L)	6.73 ± 3.92	4.69 ± 1.29	-3.343^a^	**0.001**
UCB (µmol/L)	10.40 ± 5.84	9.59 ± 3.10	-0.211^a^	0.833
TC (mmol/L)	4.33 ± 0.90	4.80 ± 0.85	3.683	**< 0.001**
TG (mmol/L)	1.01 ± 0.43	1.79 ± 1.91	-4.570^a^	**< 0.001**
HDL-C (mmol/L)	1.17 ± 0.27	1.20 ± 0.31	0.712	0.477
LDL-C (mmol/L)	2.19 ± 0.63	2.67 ± 0.77	-4.400^a^	**< 0.001**
FBG (mmol/L)	4.74 ± 0.76	5.12 ± 0.84	-3.561^a^	**< 0.001**
BPRS total score	49.80 ± 9.77	-		
Affect subscale score	7.94 ± 2.93	-		
Negative symptoms subscale score	10.79 ± 3.16	-		
Positive symptoms subscale score	12.96 ± 3.69	-		
Resistance subscale score	12.11 ± 4.28	-		
Activation subscale score	6.00 ± 2.90	-		

*n*: sample size; *P*: probability; %: percent; kg/m^2^: kilograms per square meter; µmol/L: micromoles per liter; mmol/L: millimoles per liter; TB: total bilirubin; CB: conjugated bilirubin; UCB: unconjugated bilirubin; BMI: body mass index; TC: total cholesterol; TG: triglyceride; HDL-C: high-density lipoprotein cholesterol; LDL-C: low-density lipoprotein cholesterol; FBG: fasting blood glucose; BPRS: Brief Psychiatric Rating Scale; Bolded *P* values < 0.05. a: Mann-Whitney *U* test

### Comparisons between patients with schizophrenia and healthy controls

As shown in Table 1, the patients with schizophrenia had a higher CB level, and lower levels of TC, TG, LDL-C, and FBG than HCs. Analysis of covariance (ANCOVA) showed that differences in levels of CB (F_(1,189)_ = 20.223, *P* < 0.001), TC (F_(1,189)_ = 10.380, *P* = 0.002), TG (F_(1,189)_ = 6.648, *P* = 0.011), LDL-C (F_(1,189)_ = 18.098, *P* < 0.001), and FBG (F_(1,189)_ = 6.053, *P* = 0.015), between the two groups remained significant after controlling for BMI.

### Comparisons between patients with low and high bilirubin levels

As shown in Table 2, the patients in the high TB group had a lower TG level, and a higher total score of BPRS, and higher scores on negative symptoms and resistance subscales than those in the low TB group (all *P* < 0.05). The patients in the high CB group had lower levels of TC, TG, LDL-C, a higher total score of BPRS, and scores on resistance and activation subscales than those in the low CB group (all *P* < 0.05). In addition, the patients in the high UCB group had a lower TG level, a higher HDL-C level, and a higher total score of BPRS, and a higher resistance subscale score than those in low UCB group (all *P* < 0.05).

**Table 2:** Socio-demographic and clinical characteristics of patients in low and high bilirubin groups

Variables	TB (categorical)				CB (categorical)				UCB (categorical)			
	Low group (5.9–14.3 µmol/L)	High group (14.4–56.1 µmol/L)	t/Z/χ2	*P*	Low group (1.9-5.0 µmol/L)	High group (5.1–24.2 µmol/L)	t/Z/χ2	*P*	Low group (1.7-9.0 µmol/L)	High group (9.1–39.1 µmol/L)	t/Z/χ2	*P*
Age (years)	37.51 ± 13.96	40.36 ± 13.53	-0.636	0.526	38.58 ± 14.29	39.27 ± 13.33	-0.237	0.813	37.49 ± 13.87	40.39 ± 13.62	-0.994	0.323
Male (%)	19 (42.2%)	19 (43.2%)	0.008	0.927	18 (40.0%)	20 (45.5%)	0.271	0.603	20 (44.4%)	18 (40.9%)	0.114	0.736
BMI (kg/m^2^)	21.95 ± 1.82	21.80 ± 1.65	0.452	0.652	22.08 ± 1.72	21.66 ± 1.74	1.138	0.258	21.94 ± 1.79	21.81 ± 1.68	0.361	0.719
Age of onset (years)	33.39 ± 14.05	34.75 ± 14.25	-0.048	0.962	34.13 ± 14.43	33.99 ± 13.90	0.045	0.964	33.41 ± 13.94	34.73 ± 14.37	-0.439	0.662
Duration of illness (months)	49.42 ± 72.36	65.40 ± 99.18	-0.917^a^	0.359	51.43 ± 85.35	63.35 ± 88.31	-0.884^a^	0.377	48.93 ± 72.65	65.90 ± 98.88	-1.069^a^	0.285
TC (mmol/L)	4.32 ± 0.75	4.34 ± 1.03	0.209	0.835	4.54 ± 0.83	4.12 ± 0.93	2.223	**0.029**	4.23 ± 0.79	4.43 ± 0.99	-1.115	0.268
TG (mmol/L)	1.12 ± 0.50	0.89 ± 0.32	2.406	**0.019**	1.10 ± 0.50	0.91 ± 0.33	2.093	**0.040**	1.11 ± 0.50	0.90 ± 0.33	2.288	**0.025**
HDL-C (mmol/L)	1.10 ± 0.20	1.24 ± 0.32	-1.616	0.110	1.16 ± 0.28	1.18 ± 0.27	-0.314	0.754	1.10 ± 0.20	1.25 ± 0.31	-2.716	**0.008**
LDL-C (mmol/L)	2.20 ± 0.49	2.18 ± 0.74	0.306	0.760	2.33 ± 0.54	2.05 ± 0.68	2.104	**0.038**	2.15 ± 0.52	2.23 ± 0.72	-0.657	0.513
FBG (mmol/L)	4.78 ± 0.76	4.69 ± 0.77	0.617	0.539	4.89 ± 0.75	4.58 ± 0.75	1.966	0.052	4.78 ± 0.81	4.69 ± 0.72	0.515	0.608
BPRS total score	46.46 ± 8.44	53.37 ± 9.93	-3.540	**0.001**	47.20 ± 9.00	52.45 ± 9.92	-2.619	**0.010**	46.96 ± 8.55	52.70 ± 10.18	-2.888	**0.005**
Affect subscale score	7.60 ± 3.08	8.30 ± 2.77	-1.120	0.226	7.49 ± 3.23	8.41 ± 2.55	-1.490	0.140	7.87 ± 3.04	8.02 ± 2.86	-0.250	0.803
Negative symptoms subscale score	10.09 ± 2.78	11.50 ± 3.39	-2.149	**0.034**	10.33 ± 2.99	11.25 ± 3.30	-1.375	0.173	10.29 ± 3.09	11.30 ± 3.19	-1.513	0.134
Positive symptoms subscale score	12.64 ± 3.44	13.27 ± 3.95	-0.801	0.425	13.16 ± 3.61	12.75 ± 3.80	0.516	0.607	12.58 ± 3.53	13.34 ± 3.86	-0.975	0.332
Resistance subscale score	10.62 ± 3.68	13.64 ± 4.35	-3.533	**0.001**	10.96 ± 3.95	13.30 ± 4.32	-2.668	**0.009**	10.58 ± 3.60	13.68 ± 4.38	-3.654	**< 0.001**
Activation subscale score	5.42 ± 2.23	6.59 ± 3.39	-1.919	0.059	5.27 ± 2.37	6.75 ± 3.22	-2.470	**0.016**	5.64 ± 2.29	6.36 ± 3.42	-1.165	0.248

*P*: probability; %: percent; kg/m^2^: kilograms per square meter; µmol/L: micromoles per liter; mmol/L: millimoles per liter; TB: total bilirubin; CB: conjugated bilirubin; UCB: unconjugated bilirubin; BMI: body mass index; TC: total cholesterol; TG: triglyceride; HDL-C: high-density lipoprotein cholesterol; LDL-C: low-density lipoprotein cholesterol; FBG: fasting blood glucose; BPRS: Brief Psychiatric Rating Scale. Bolded *P* values < 0.05. a: Mann-Whitney *U* test

### Factors associated with high bilirubin levels (categorical)

Binary logistic regression (model 1: BPRS total score, or model 2: BPRS subscale scores involved in the regression models, respectively) analyses revealed that the high TB level (categorical) was significantly associated with a lower TG level [model 1: Odds Ratio (OR) = 0.25, 95% Confidence Interval (CI) = 0.07–0.90; model 2: OR = 0.25, 95% CI = 0.07–0.87], a higher total score of BPRS (OR = 1.09, 95% CI = 1.03–1.16) and a higher resistance subscale score (OR = 1.17, 95% CI = 1.04–1.32). The high CB level was significantly associated with a lower TC level, a higher total score of BPRS and a higher resistance subscale score (all *P* < 0.05). And the high UCB level was significantly associated with a higher HDL-C level, a higher total score of BPRS, and a higher resistance subscale score (all *P* < 0.05) (Table 3).

**Table 3:** Independent factors associated with bilirubin levels in patients

Variables	Model 1				Model 2			
	*P*	OR	95%CI		*P*	OR	95%CI	
			Lower	Upper			Lower	Upper
**TB (categorical)**								
TG (mmol/L)	**0.033**	0.251	0.070	0.895	**0.030**	0.248	0.070	0.873
BPRS total score	**0.006**	1.090	1.025	1.160	-	-	-	-
Resistance subscale score	-	-	-	-	**0.009**	1.173	1.041	1.322
**CB (categorical)**								
TC (mmol/L)	**0.015**	0.477	0.263	0.867	**0.008**	0.434	0.234	0.802
BPRS total score	**0.013**	1.071	1.015	1.131	-	-	-	-
Resistance subscale score	-	-	-	-	**0.005**	1.185	1.053	1.335
**UCB(categorical)**								
HDL-C (mmol/L)	**0.049**	6.540	1.006	42.505	0.064	6.103	0.902	41.296
BPRS total score	**0.019**	1.072	1.012	1.135	-	-	-	-
Resistance subscale score	-	-	-	-	**0.006**	1.187	1.051	1.338

*P*: probability; mmol/L: millimoles per liter; TB: total bilirubin; CB: conjugated bilirubin; UCB: unconjugated bilirubin; TG: triglyceride; TC: total cholesterol; HDL-C: high-density lipoprotein cholesterol; BPRS: Brief Psychiatric Rating Scale; OR: odds ratio; CI: confidence interval. Bolded *P* values < 0.05

Model 1: BPRS total score involved in the regression models

Model 2: BPRS subscale scores involved in the regression models

### Independent correlates of bilirubin levels (continuous)

There were several correlations between socio-demographic and clinical variables with bilirubin levels (continuous) in patients (Table 1S). Multivariate linear regression analyses showed that the TB level was negatively associated with levels of TG (*β* = -0.26, *P* = 0.025), and was positively associated with resistance subscale score (*β* = 0.05, *P* < 0.001). The CB level was negatively associated with levels of TG and FBG, and was positively associated with total score of BPRS (all *P* < 0.05). The UCB level was negatively associated with TG level, and was positively associated with resistance subscale score (all *P* < 0.05) (Table 2S).

## Discussion

In this study, we examined the peripheral bilirubin levels and their associations with psychopathology and glycolipid metabolism parameters in patients with AEDF schizophrenia. Consistent with the findings of two recent studies conducted in patients with acute-episode schizophrenia, we found that patients with AEDF schizophrenia had a higher CB level than HCs [23, 31]. This may be due to the fact that patients with acute onset schizophrenia are in a state of high oxidative stress, and sustained oxidative stress induces rupture of the erythrocyte cell membrane, which increases serum bilirubin levels [32]. Since CB is formed by the conversion of UCB in the liver, a significant elevated CB level may reflect an increased overall bilirubin level. In turn, elevated UCB levels induce reactive oxygen species (ROS) and nitric oxide (NO) production by neuroglia cells, leading to further enhancement of oxidative stress [33]. In a previous study, drug-naive, first-episode patients with schizophrenia had significantly higher levels of UCB, compared to HCs [34]. A new insight suggests that we can distinguish schizophrenia from schizoaffective and bipolar disorders based on the degree of UCB elevation [35, 36]. However, there were no differences in UCB levels between patients with schizophrenia and HCs in this study. On the contrary, in a recent study, Huang et al. reported that there was no statistical difference in bilirubin levels between patients with first-episode schizophrenia and HCs [37]. Even more, some studies conducted in different countries (South Korea, China, and Norway) found varying degrees of reduction in serum bilirubin levels in patients with schizophrenia compared with HCs [21, 38, 39]. In this case, reduced bilirubin levels may indicate a defect in the antioxidant defense system of patients with schizophrenia or a sustained oxidative depletion during the chronic course of this disease [40, 41].

In this study, we also found some associations between bilirubin and psychopathology in patients with AEDF schizophrenia. First, bilirubin levels were positively associated with the total score of BPRS. A clinical study found that increased oxidative metabolites of bilirubin are correlated with higher scores of BPRS in schizophrenia, and higher scores of Hamilton depression scale (HAMD) in depression [42]. These findings suggest that higher bilirubin levels are associated with exacerbated psychotic states, possibly as a result of bilirubin-induced neurotoxic effects and pro-inflammatory responses, as well as alterations in brain structures. For example, high bilirubin levels result in a rapid increase in extracellular glutamate concentrations, which ultimately leads to neuronal and oligodendrocyte damage [43]. In addition, by inducing the secretion of tumor necrosis factor-α (TNF-α) and interleukin-1β (IL-1β), bilirubin provokes mitochondrial swelling, and inhibits the formation of myelin, which aggravates the patient’s psychiatric symptoms [44–46]. Neuroimaging studies found that patients with schizophrenia combined with hyperbilirubinemia have an enlarged cerebrospinal fluid cavity and enhanced signal in areas such as the frontotemporal cortex and the limbic system and basal ganglia [47–49].

Second, we found that bilirubin levels were positively associated with the BPRS resistance subscale score, which is usually defined by hostility, uncooperativeness and suspiciousness [30]. One Portuguese study [24] found that a positive association between UCB level with disturbing and aggressive behaviors in patients with schizophrenia and schizoaffective, which bring a new insight into the novel role of UCB level as a biomarker for psychomotor agitation. This was confirmed by the improvement in psychomotor agitation with decreasing bilirubin levels during a subsequent treatment. In patients with hyperbilirubinemia, elevated oxygen radicals or oxidative stress markers are associated with an increased risk of aggressive behaviors [50, 51]. Sustained oxidative stress can lead to reduced neuronal membrane fluidity, ion channel inactivation and demyelination, which may result in brain connectivity and impulse control disorders [52, 53]. A meta-analysis and the United States National Survey showed that the prevalence of aggressive behavior in patients with schizophrenia and bipolar disorder was 33.3% and 12.2%, respectively, both much higher than in the general population [54, 55]. Therefore, it is necessary to monitor bilirubin levels, which can objectively and accurately assess the risk of aggression and hostility in patients with severe mental illness. Third, a study conducted in unmedicated patients with schizophrenia found that bilirubin levels were positively correlated with the excitement component score of Positive and Negative Syndrome Scale (PANSS) [22]. Similarly, we also found a significant correlation between a higher CB level and a higher activation subscale score of BPRS, which is usually defined by excitement, tension, and mannerisms–posturing. However, the correlation disappeared after adjusting for the other BPRS subscale scores. Our interpretation is that the high CB level may be an indicator of a higher activation score, but may not be independent of the other BPRS subscale scores. In clinical evaluation, the use and dissemination of biomarkers that can provide a mode of clinical monitoring are the future frontiers in the evaluation of schizophrenia and related spectrum disorders. In therapy, anti-inflammatory drugs were considered an effective and safe adjunctive therapy for improving the symptoms of schizophrenia [56]. For example, as an anti-inflammatory drug, minocycline can reduce oxidative stress and protect the brain from bilirubin neurotoxicity [57].

Overall, we also found that bilirubin levels were negatively associated with levels of FBG, TG, and TC, and were positively associated with HDL-C level in patients with AEDF schizophrenia. Of note, we observed differences in glycolipid metabolic parameters in patients with different hyperbilirubinemia subtypes. Specifically, in the logistic regression analysis model, patients with a higher TB level had a lower TG level, those with a higher CB level had a lower TC level, and those with a higher UCB level had a higher HDL-C level; In the linear regression model, CB, but not UCB levels, were negatively correlated with the FBG level. The differences were also observed in the general population and schizophrenia patients in previous cross-sectional and prospective studies [58, 59]. Bilirubin is a lipid-soluble substance, and different types of bilirubin bind differently to albumin. Compared to UCB, CB may be more readily separated from albumin to protect patients from metabolism disorders [17]. Regarding the mechanism of action of bilirubin on glycolipid metabolism, it has been found that bilirubin can reduce glucose and lipid accumulation by increasing insulin sensitivity and intracellular glucose uptake, and activating the aryl hydrocarbon receptor (AhR) signaling pathway [60, 61]. A meta-analysis found that the overall prevalence of MetS in patients with schizophrenia was 32.5%, and the prevalence in unmedicated patients was also as high as 20.2% [62]. Notably, the risk of cardiovascular death in patients with MetS was 1.6 times higher than in those without, and 3.2 times higher than in the general population [63, 64]. As a valid predictor of MetS risk, bilirubin has potential use in monitoring and assessing the risk of cardiovascular events and death in patients with schizophrenia.

In this study, there are several limitations. First, due to methodological limitations, including cross-sectional study design, no patients with medicated schizophrenia or other severe mental disorders (e.g. schizoaffective and bipolar disorders), we were unable to make comparisons of bilirubin levels between subgroups [65]. Second, factors potentially associated with bilirubin levels, such as smoking, the use/abuse of caffeine, and pro re nata treatment (e.g. psychotherapy and counseling intervention) before blood sampling, were not examined. Finally, we have to acknowledge the importance of excluding pseudo- or organic schizophrenia, such as Gilbert syndrome by genetic testing, encephalitis by cerebrospinal fluid testing, and encephalic anomaly by electroencephalography and magnetic resonance imaging [24, 66]. Therefore, our results should be considered preliminary.

## Conclusion

In summary, patients with AEDF schizophrenia had higher bilirubin levels, especially CB level, compared with HCs. Patients with high bilirubin levels have an exacerbated psychotic state with high risk of aggression, hostility and excitement; and have relatively optimized glycolipid metabolism with lower TG, TC and FBG levels and higher HDL-C levels. In clinical practice, regular monitoring of bilirubin levels is necessary to objectively and comprehensively assess psychopathology and the risk of glycolipid metabolism disorders in patients with schizophrenia. Given that our findings are concerning but preliminary, longitudinal studies are needed to further explore whether management of peripheral bilirubin could improve the prognosis of this patient population.

### Electronic supplementary material

Below is the link to the electronic supplementary material.

Supplementary Material 1

## Data Availability

The data that support the findings of this study are available from the corresponding author upon reasonable request.
