# Supplementary material for: The associations of psychopathology and metabolic parameters with serum bilirubin levels in patients with acute-episode and drug-free schizophrenia: a 5-year retrospective study using an electronic medical record system
Source: BMC Psychiatry. 2024 May 29;24:403. doi: 10.1186/s12888-024-05862-5 (PMC11138041; doi:10.1186/s12888-024-05862-5)
Supplement: Supplementary file 1 — Supplementary Material 1 [file 12888_2024_5862_MOESM1_ESM.docx]

**Supplementary Appendix**

**Table of Contents**

**Supplementary Table 1** Correlations between socio-demographic and clinical variables with bilirubin levels (continuous) in patients.

**Supplementary Table 2** Independent correlates of bilirubin levels (continuous) in patients.

**Supplementary Table 1** Correlations between socio-demographic and clinical variables with bilirubin levels (continuous) in patients.

| **Variables** | **TB (continuous)** | | **CB (continuous)** | | **UCB (continuous)** | |
| --- | --- | --- | --- | --- | --- | --- |
|  | ***r*** | ***P*** | ***r*** | ***P*** | ***r*** | ***P*** |
| Age (years) | 0.010 | 0.928 | -0.074 | 0.489 | 0.043 | 0.689 |
| Male（%） | 0.003 | 0.981 | 0.013 | 0.907 | -0.006 | 0.956 |
| BMI (kg/m2) | -0.113 | 0.293 | -0.068 | 0.524 | -0.128 | 0.233 |
| Age of illness onset (years) | -0.037 | 0.731 | -0.085 | 0.429 | -0.038 | 0.725 |
| Duration of illness (months) | 0.120 | 0.262^a^ | 0.058 | 0.588^a^ | 0.163 | 0.127^a^ |
| TC (mmol/L) | -0.055 | 0.608 | -0.241 | **0.023** | 0.043 | 0.686 |
| TG (mmol/L) | -0.272 | **0.010** | -0.285 | **0.007** | -0.281 | **0.008** |
| HDL-C (mmol/L) | 0.195 | 0.066 | 0.105 | 0.326 | 0.236 | **0.026** |
| LDL-C (mmol/L) | -0.115 | 0.285 | -0.271 | **0.010** | 0.009 | 0.993 |
| FBG (mmol/L) | -0.246 | **0.020** | -0.297 | **0.005** | -0.159 | 0.136 |
| BPRS total score | 0.420 | **< 0.001** | 0.432 | **< 0.001** | 0.393 | **< 0.001** |
| Affect subscale score | -0.004 | 0.969 | 0.032 | 0.767 | -0.054 | 0.617 |
| Negative symptoms subscale score | 0.286 | **0.007** | 0.305 | **0.004** | 0.289 | **0.006** |
| Positive symptoms subscale score | 0.130 | 0.225 | 0.117 | 0.275 | 0.151 | 0.158 |
| Resistance subscale score | 0.450 | **< 0.001** | 0.412 | **< 0.001** | 0.459 | **< 0.001** |
| Activation subscale score | 0.278 | **0.008** | 0.335 | **0.001** | 0.193 | 0.069 |
| *P*: probability; %: percent; kg/m^2^: kilograms per square meter; mmol/L: millimoles per liter; TB: total bilirubin; CB: conjugated bilirubin; UCB: unconjugated bilirubin; BMI: body mass index; TC: total cholesterol; TG: triglyceride; HDL-C: high-density lipoprotein cholesterol; LDL-C: low-density lipoprotein cholesterol; FBG: fasting blood glucose; BPRS: Brief Psychiatric Rating Scale. Bolded *P* values < 0.05. a: Spearman correlation analysis | | | | | | |

**Supplementary Table 2** Independent correlates of bilirubin levels (continuous) in patients.

| **Variables** | **B** | **SE** | ***β*** | ***P*** |
| --- | --- | --- | --- | --- |
| **TB (continuous)** | | | | |
| TG (mmol/L) | -0.257 | 0.112 | -0.226 | **0.025** |
| Resistance subscale score | 0.051 | 0.011 | 0.444 | **< 0.001** |
|  |  |  |  |  |
| **CB (continuous)** | | | | |
| TG (mmol/L) | -0.220 | 0.118 | -0.184 | 0.065 |
| FBG (mmol/L) | -0.152 | 0.067 | -0.222 | **0.027** |
| BPRS total score | 0.022 | 0.005 | 0.410 | **< 0.001** |
|  |  |  |  |  |
| **UCB (continuous)** | | | | |
| TG (mmol/L) | -0.267 | 0.120 | -0.222 | **0.029** |
| Resistance subscale score | 0.053 | 0.012 | 0.430 | **< 0.001** |
| *P*: probability; mmol/L: millimoles per liter; TB: total bilirubin; CB: conjugated bilirubin; UCB: unconjugated bilirubin; TG: triglyceride; FBG: fasting blood glucose; SE: standard error. Bolded *P* values < 0.05. | | | | |
